# Pine wilt disease: what do we know from proteomics?

**DOI:** 10.1186/s12870-024-04771-9

**Published:** 2024-02-09

**Authors:** Joana M. S. Cardoso, Bruno Manadas, Isabel Abrantes, Lee Robertson, Susana C. Arcos, Maria Teresa Troya, Alfonso Navas, Luís Fonseca

**Affiliations:** 1 Centre for Functional Ecology, Associate Laboratory TERRA, Department of Life Sciences, University of Coimbra, Calçada Martins de Freitas, Coimbra, 3000-456 Portugal; 2 CNC-Center for Neuroscience and Cell Biology, University of Coimbra, Rua Larga, Polo I, Coimbra, 3004-504 Portugal; 3 CIBB - Centre for Innovative Biomedicine and Biotechnology, University of Coimbra, Rua Larga - Faculdade de Medicina, 1ºandar - POLO I, Coimbra, 3004-504 Portugal; 4 Instituto Nacional de Investigación y Tecnología Agraria y Alimentaria, CSIC. Instituto de Ciencias Forestales (ICIFOR), Ctra. de La Coruña Km 7.5, Madrid, 28040 Spain; 5 Museo Nacional de Ciencias Naturales, CSIC. Dpto Biodiversidad y Biología Evolutiva, C/ José Gutiérrez Abascal 2, Madrid, 28006 Spain

**Keywords:** Biomarkers, Pine trees, Pine wood nematode, Plant-pathogen interactions, Proteomics

## Abstract

Pine wilt disease (PWD) is a devastating forest disease caused by the pinewood nematode (PWN), *Bursaphelenchus xylophilus*, a migratory endoparasite that infects several coniferous species. During the last 20 years, advances have been made for understanding the molecular bases of PWN-host trees interactions. Major advances emerged from transcriptomic and genomic studies, which revealed some unique features related to PWN pathogenicity and constituted fundamental data that allowed the development of postgenomic studies. Here we review the proteomic approaches that were applied to study PWD and integrated the current knowledge on the molecular basis of the PWN pathogenicity. Proteomics has been useful for understanding cellular activities and protein functions involved in PWN-host trees interactions, shedding light into the mechanisms associated with PWN pathogenicity and being promising tools to better clarify host trees PWN resistance/susceptibility.

## Background- pine wilt disease

The expansion of global trade activities within global warming scenarios have enhanced the pests and pathogens dissemination to non-infected areas contributing to several epidemic events [1, 2]. Plant-parasitic nematodes are among the most widespread and damaging global pests in agronomy and forestry and one of the top 10 plant-parasitic nematodes with the highest global economic and ecological importance is the pinewood nematode (PWN), *Bursaphelenchus xylophilus*, classified as a A2 quarantine organism by the European Plant Protection Organisation [3]. This migratory endoparasite nematode is considered the causal agent of the Pine wilt disease (PWD), a complex disease caused by tripartite species interactions: *B. xylophilus*, the causal organism; *Pinus* spp., the host tree; and *Monochamus* spp., the insect vector [4].

The PWN is indigenous to North America, where it poses little threat to the native conifer trees, causing disease only on a few exotic pine species [4]. At the beginning of the twentieth century, it spread to Japan [5], China [6], Taiwan [7] and South Korea [8] where it has been responsible for the devastation of enormous pine forests areas. In 1999, it was reported, for the first time in Europe, in continental Portugal [9] and later in Spain [10, 11] and Madeira Island [12].

The genus *Bursaphelenchus* includes nematodes distributed for Africa, America, Asia, and Europe with more than 100 valid species [13]. Most of the species of this genus are mycophagous, have a phoretic relationship with insects, mainly bark beetles and wood borers belonging to the Scolytidae, Cerambycidae, Curculionidae, and Buprestidae families, and have been associated with dead or dying conifers [4]. In the case of the PWN the most important vectors are cerambycids beetles of the *Monochamus* genus. In North America, the most important vector is *M. carolinensis* [14], while in Asia is *M. alternatus* [15]. In Portugal [16] and Spain [17], *M. galloprovincialis* is the only identified vector.

The main host plants of *B. xylophilus* are tree species belonging to the genus *Pinus*, but the list of susceptible plants also includes other coniferous species of the genera *Abies*, *Chamaecyparis*, *Cedrus*, *Larix*, *Picea* and *Pseudotsuga* [4, 18]. The most susceptible European species of the genus *Pinus* is the Scotch pine, *P. sylvestris,* widespread throughout central and northern Europe. Other highly susceptible species are: *P. pinaster*, the maritime pine; *P. mugo*, the dwarf mountain pine or mountain pine; and *P. nigra*, the black pine [18]. In Europe, the PWN was reported in Portugal associated with *P. pinaster* [9, 12] and *P. nigra* [19] and in Spain, associated with *P. pinaster* [10, 11] and *P. radiata* [20].

**Fig. 1:**
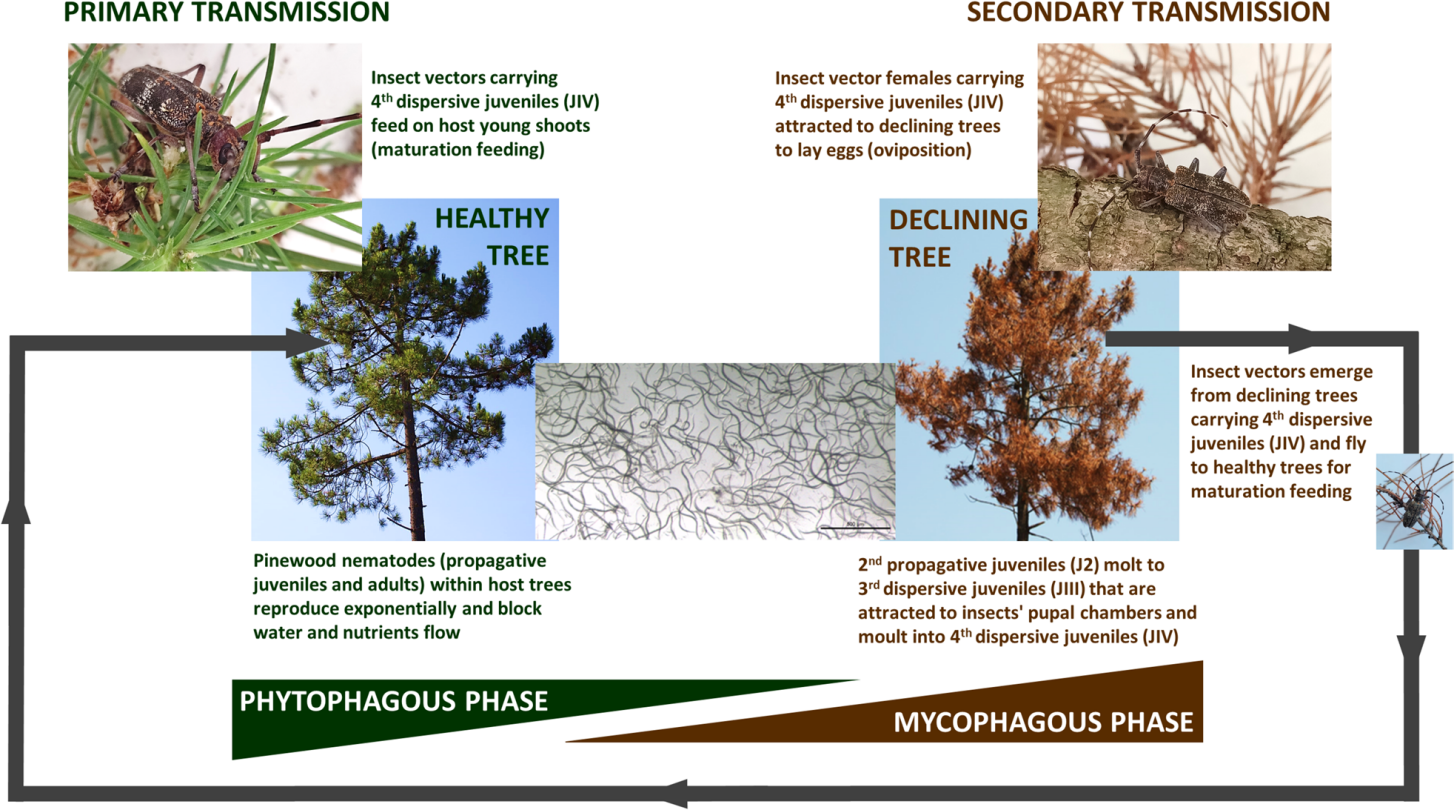
Pine wilt disease species interactions among the pinewood nematode, *Bursaphelenchus xylophilus*, a host tree, *Pinus pinaster* and an insect vector, *Monochamus galloprovincialis*

The PWN life cycle can have two phases, phytophagous and mycophagous (Fig. 1). In the phytophagous phase, nematodes feed on live cells of the host trees while in the mycophagous phase they feed on fungi of declining host trees [4, 21, 22]. Both phases can comprise four propagative juvenile stages (J1- inside the eggs, J2, J3 and J4) and adults with sexual dimorphism. When environmental conditions become unfavorable, nematodes experience periods of desiccation or food shortage. During this time, the propagative stage (J2) undergoes significant morphological and physiological changes, leading to the emergence of the third dispersive juvenile stages (JIII) and then to the fourth dispersive juvenile stage (JIV) [21, 23, 24]. The JIII, known as pré-dauer juveniles, considered the most resistant stage, are characterized by having a thick cuticle, a well-defined head region, a rounded tail terminus and high lipid content in the intestine by deposition of lipid droplets. The JIV, also known as dauer juveniles, are mobile, do not feed, have a thicker basal layer, and have an external cortical layer with large lipid droplets. They also do not have stylet, esophagus, and esophageal glands. The tail is sub-cylindrical, with a digitate terminus and are well adapted to be carried by the insect vector by having a protective adhesive substance covering the cuticle [4, 24].

When the insect vector carrying nematodes (JIV) emerges from dead or declining pine trees, they immediately fly to healthy trees and feed on young shoots (maturation feeding) causing feeding wounds (primary transmission) (Fig. 1). These wounds correspond to entry portals for the nematode. After entering the tree, the nematodes molt to adults and start reproducing exponentially and three to four weeks after infection, the host trees begin to display wilting symptoms (Fig. 1). In infected plants, there is a cessation of resin exudation due to the rupture of the resin canals and the dissemination of oleoresins into adjacent tracheids causing cavitation and embolism [4, 24]. Other symptoms associated with the PWN infection are the yellowing and wilting of needles until they reach a red-brown shade, partial or total dryness of the crown and, in a more advanced stage, the existence of brittle branches. These symptoms are caused by a reduction in the translocation of water and solutes and become visible mainly in the period of late summer and early autumn. The intensity of the symptoms observed varies according to the host plant species, temperature and time of the year [18, 23]. Declining or dead trees, already invaded by fungi, may contain millions of nematodes, creating favorable conditions for the mycophagous phase. Female insects use these declining trees for oviposition (secondary transmission) (Fig. 1). After laying the eggs, the insect pupates in the pupal chambers and later the pupae enclose in the chamber and remains there for several days as a callow adult, during which PWN JIV enter the vector’s body. Then, the new insect emerges carrying nematodes mainly in their tracheal system and feeds on young shoots of new host plants and transmits the nematodes to a new host plant (Fig. 1) [4, 24, 25].

The susceptibility/resistance of trees differs between *Pinus* species (interspecific variability) and among trees of the same species (intraspecific variability). Numerous physiological, histological, structural, and biochemical studies have been performed to determine the mechanism of symptomatology development. A series of chemical changes, such as the production and accumulation of ethylene and condensed tannins (proanthocyanidins) responsible for tissue browning, generation of superoxide anions, vacuoles in ray parenchyma cells, increase in lipid peroxidation, electrolytes leaking from cells, emission of volatiles and accumulation of phytotoxic substances, may occur in infected trees, accelerating the development of the disease [26, 27]. In some cases, host cells death and symptoms progression is so fast that some authors have mentioned that PWN may produce phytotoxins responsible for the cell death in the host [28, 29]. However, the role of these toxins in symptom development and their origin has not been clarified, and some studies have proposed that these toxins could have an origin in bacteria [30–34]. Moreover, various metabolites such as terpenoids [35, 36] and phytoalexin [37], stilbenoids [38] and flavonoids [38], related to the plant resistance mechanisms, may also accumulate in the trees and some revealed nematicidal activity [39, 40].

To study the PWN pathogenicity, biological, behavioral, reproductive, physiological, and molecular traits that influence the pathogenicity status were compared between PWN and nonpathogenic species, mainly *B. mucronatus*, the closest related non-pathogenic species under laboratory conditions, indicating that *B. xylophilus* had a higher developmental, population growth and dispersing rates [41–44]. Furthermore, depending on the host plant, geographical isolation, and environmental stress, PWN pathogenicity status varies greatly among isolates. In order to evaluate and characterize virulent and avirulent isolates, several analyses have been made on isozyme profiles [42], in vivo and in vitro reproductive capacity [45], dispersion and feeding ability in pine tissues [46], JIII development rate into JIV in the insect pupal chamber [47], oxidative stress tolerance [48, 49] and pathogenesis-related genes sequencing [50, 51].

## *Bursaphelenchus xylophilus* transcriptomics and genomics

During the last 20 years, advances have been made in understanding the molecular bases of PWN-host interactions and pathogenic mechanisms, and these advances emerged mainly from transcriptomics and genomics on *B. xylophilus*.

The first key steps towards the molecular dissection on *B. xylophilus* parasitism date from 2007 with a large-scale expressed sequence tag (EST) project [52] followed by the first *B. xylophilus* draft genome in 2011 with 74.6 Mbp of assembled sequence [53]. Several other studies, based on *B. xylophilus* transcriptome, became a good contribution to clarify its pathogenicity, allowing the identification of several pathogenesis-related genes (e.g., glycosyl hydrolase family 45 cellulases, endo-β-1,3-glucanase, pectate lyases, pectidases, peroxiredoxin, chitinases, calreticulin, venom allergen proteins), involved in several cellular processes like cell wall degradation, feeding, detoxification and reproduction [54–60] and also providing information on its evolutionary origin [61]. Some of these pathogenesis-related genes have been suggested as acquired from bacteria and fungi by horizontal gene transfer processes [53]. Additionally, few other genomic studies contributed to a better understanding of evolutionary ecology and different pathogenicity among *B. xylophilus* isolates [50, 62, 63] and more recently, a nearly complete *B. xylophilus* genome sequence has become available with a final assembly of 78.3 Mbp long [64].

All these transcriptomics and genomics studies have contributed to better understanding of this nematode’s biology and have provided fundamental data that permitted the development of the first *B. xylophilus* proteomics studies in the last decade [65].

In addition to comparative transcriptomic and genomic analyses, molecular characterisation of proteins belonging to different families such as cysteine peptidases, aspartic peptidases, α-l-fucosidases, fatty acid-and retinol-binding proteins, cystatins, calreticulins, peroxiredoxins, heat shock proteins, venome allergen proteins, has been performed based on gene sequencing and in silico protein sequence and 3D structural analyses, ligand binding ability and immunolocalization in pine stems [66–72]. Functional genomics with several PWN gene silencing by RNA interference (RNAi) has also been applied [73–81]. The application of this technique, as a tool for the genes functional analysis has contributed to the identification of PWN genes with metabolic functions in its development, multiplication, survival and parasitism. However, the efficacy and reproducibility of RNAi in *B. xylophilus* vary widely depending on the target gene, expression localization and soaking conditions, being difficult to validate a candidate as pathogenicity factor by RNAi gene silencing techniques [73, 74, 82]. CRISPR/Cas9 is a powerful experimental tool for gene-editing [83] and is clearly an emerging tool for functional genomics, opening new opportunities for functional analysis in many nematodes, including *B. xylophilus* [82, 84].

Additionally, some transcriptomics studies on *B. xylophilus* host trees have been published. Comparative analysis of transcriptomes of *Pinus* species with different susceptibility to *B. xylophilus* infection revealed different strategies for handling nematode infection [85–89]. *Pinus pinaster* presented a higher abundance of genes related to transcriptional regulation, terpenoid secondary metabolism (including some with nematicidal activity) and pathogen attack. *Pinus pinea* showed a higher abundance of genes related to oxidative stress and higher levels of expression of stress-responsive genes [85]. Moreover, Modesto and co-authors reviewed several works on the molecular defense response of pine trees after infection with PWN. This overview highlighted several common pathways associated with resistance in the different pine species or varieties, including activation of ROS detoxification, cell wall lignification, and biosynthesis of terpenoids and phenylpropanoids with nematicidal effects [90]. These studies provided essential information about the molecular defense mechanisms used by several pine species against *B. xylophilus* infection, contributing to a better understanding of the pine wilt disease. Besides, these studies constitute important data for the development of pine trees proteomics studies.

## Proteomics

Proteomics comprises the range of technical approaches used to study proteomes, the high-throughput characterization of the protein content of an organism or sample, in a given time point, under specific conditions [91]. In proteomics approaches, proteins are usually digested and the small peptide sequences are identified by mass spectrometry and matched to protein sequences available in databases [92]. The abundance of each protein in a specific sample can also be determined using mass spectrometry and bioinformatics [92]. The proteome is highly variable over time, among samples and environmental changes and it is related to mRNA (transcriptome) data but dependent on translation efficiency and post-translation modifications. Comparing proteomes allows the identification of proteins that are differentially expressed in distinct cell populations or in response to different treatments. Since proteins are the final product of gene regulation and provide the final evidence of the function of a gene, proteomics studies are important complements to transcriptomics and genomics. They are fundamental in finding out which proteins are effectively produced and clarifying which molecules are directly involved in the host-parasite interaction.

### Proteomics methodologies

Proteomics methodologies aim to analyse a large number of proteins within a certain set of samples and have recently evolved due to technological advances in mass spectrometry (MS), optimization in sample preparation, and computer sciences that allow us to deal with the large amount of information generated by the MS-based technologies. These approaches can deliver different types of data, such as the identification of proteins in the sample at a given moment and the expression levels of the proteins (quantitative proteomics) [93]. The quantitative information can be acquired as an absolute quantification, where the amount of the protein in the sample is calculated, or relative quantification, where the amount of a given protein is expressed as a fold change for the same protein relative to another condition [94].

The classical approach to obtain relative quantifications of a proteome was bidimensional electrophoresis (2DE-Isoelectric focusing followed by SDS-PAGE), where the identification of the proteins was obtained by an MS analysis and the relative quantification done measuring the staining density of matched gel spots. However, in this method, some types of proteins are underrepresented, and although hundreds to a few thousands of proteins may be detected, many proteins with lower abundance are very difficult to quantify. Also, the analysis of many samples by this method is laborious and time-consuming. Therefore, several methodologies were developed over the years that support proteomic expression level quantification. In general, an MS-based proteomics experiment comprises the enzymatic digestion of the proteins, commonly using trypsin, separation of the generated peptides by reversed-phase liquid chromatography (RP-LC), and on-line mass spectrometry characterization of the eluted peptides [95]. Although the most popular LC–MS quantitative approaches used to be called labeled approaches (which require the stable isotopic labeling of the samples prior to MS analysis, such as iTRAQ or TMT), the label-free approaches gained increased interest mostly due to the higher accuracy and sensitivity of MS instruments and improvement of the algorithms for data analysis [96].

Protein identification in LC–MS label-free approaches has been dominated by data-dependent acquisition methods (DDA, also called information-dependent acquisition—IDA), where the instruments are set to scan the precursor ions followed by the selection of a limited set to be fragmented, usually the most intense ones. The fragmentation spectra (MS/MS spectra) obtained are characteristic of a given peptide and are used for its identification. While this method is particularly effective for protein identification, it presents some disadvantages that have limited its use in protein quantification between multiple samples. Therefore, the use of data-independent acquisition (DIA) methods, where fragmentation spectra are acquired for the entire sample without any pre-selection of precursor ions, started to be used for label-free quantitative approaches as an alternative to the limitations of IDA experiments [96].

Several DIA methods were developed, and the sequential window acquisition of all theoretical mass spectra (SWATH-MS) acquisition method was recognized as an unbiased method capable of quantifying a large number of peptides with consistency and accuracy constituting a good strategy for biomarker discovery from large-scale screenings [97].

The correct identification of protein and peptide sequences is fundamentally important in proteomics research and database searching is the most widely used method for peptide identification. The sequence database searching method is performed using specific software tools and a reference peptide sequence database is constructed from available protein sequences by in silico digesting them into peptides, following protease specificity rules. Peptide identification is achieved by matching the experimental spectra with the theoretical fragmentation patterns of peptides in the reference database. The database search strategy requires a robust method to assess the false discovery rate (FDR) in identification and the correct and completeness of identifications depend greatly on the availability and quality of the used databases [98], which result mainly from previous transcriptomics or genomics studies.

### *Bursaphelenchus xylophilus* proteomics

**Fig. 2:**
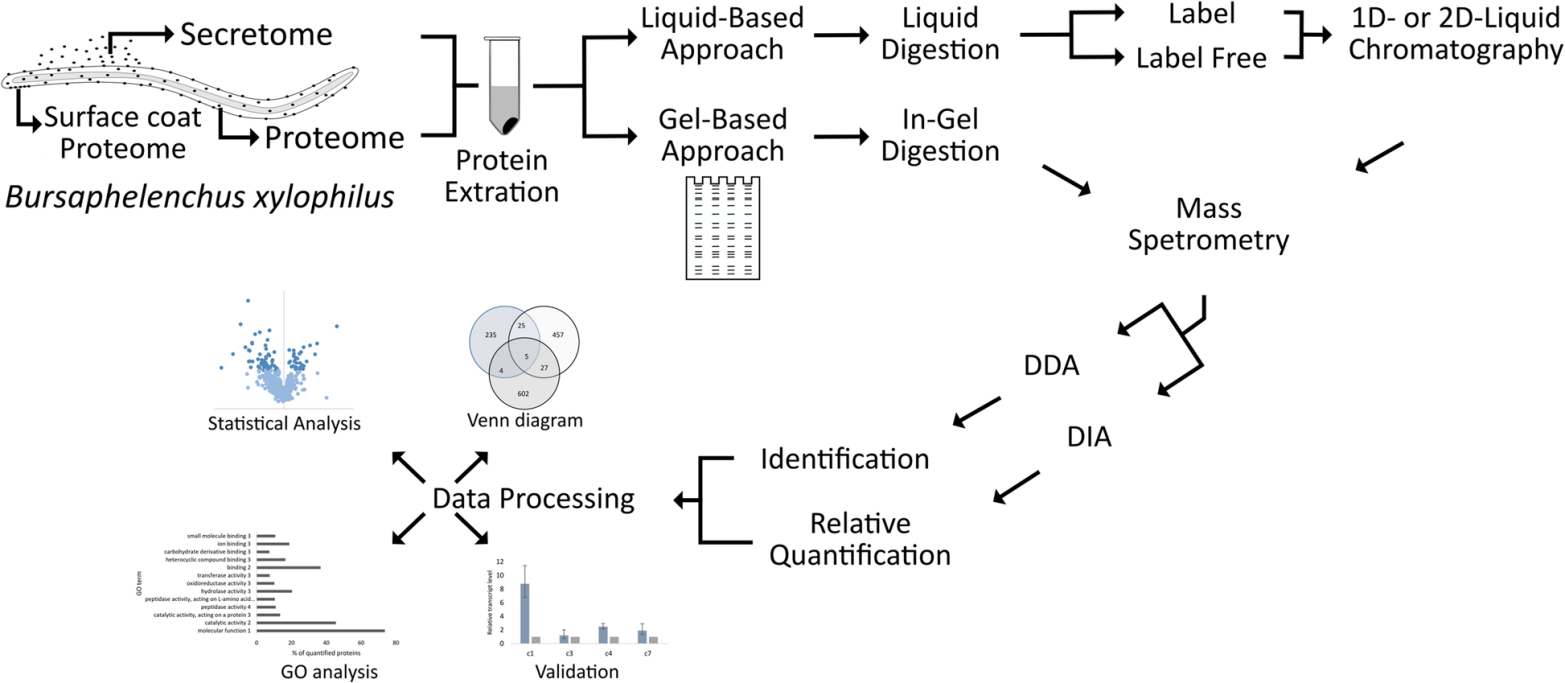
Generic experimental workflow for *Bursaphelenchus xylophilus* proteomics. Data-dependent acquisition (DDA); data-independent acquisition (DIA); gene ontology (GO)

During the last decade, few studies were published focusing on *B. xylophilus* proteomics data (Table 1). In Fig. 2, the general workflow used for *B. xylophilus* proteomes/secretomes analysis is represented.

**Table 1:** *Bursaphelenchus xylopilus* proteomics scientific publications

References	Objective	Target protein(s)	Proteomic method(s)	Protein dataset identifier
[99]	To identify differentially expressed surface coat proteins of *B. xylophilus* during host pine infection and culture in vitro	Surface coat proteins	Reversed-phase high-performance liquid chromatography (RP-HPLC) and sodium dodecyl sulfate poly-acrylamide gel electrophoresis (SDS-PAGE) followed by Matrix-assisted laser desorption ionization time-of-flight mass spectrometry analysis (MALDI-TOF/MS)	–––––
[100]	To identify the target protein of a specific monoclonal antibody; diagnosis biomarker	Target protein for a specific monoclonal antibody	2-D nano liquid chromatography electrospray ionization quadrupole ion trap tandem mass spectrometry (nano-LC-ESI-Q-IT-MS/MS); data collected in information-dependent acquisition (IDA)	–––––
[101]	To obtain *B. xylophilus* secretome profile	Secretome	Nano LC–MS/MS; data collected in IDA	–––––-
[102]	To identify population biomarkers	Proteome	Labelled iTRAQ; 2D-nano LC–ESI–MS/MS analysis; absolute quantification	PXD003129
[103]	To compare *B. xylophilus* and *B. mucronatus* secretomes; identify pathogenicity biomarkers	Secretome	Non-label short-GeLC, data collected in IDA and comparative quantitative analysis by sequential window acquisition of all theoretical mass spectra (SWATH-MS)	––––––
[104]	To compare *B. xylophilus* secretomes under pine tree stimuli with different susceptibility to PWD; identify pathogenicity biomarkers	Secretome	Non-label short-GeLC, data collected in IDA and comparative quantitative analysis by SWATH-MS	PXD024011
[105]	To compare secretomes of *B. xylophilus* isolates with different virulence; identify virulence determinants	Secretome	2D-HPLC and SDS-PAGE; comparative quantitative analysis by gel bands intensities; followed by nano LC–MS/MS of selected bands; data collected in IDA	–––––
[51]	To compare secretomes and proteomes of *B. xylophilus* isolates with different virulence; identify pathogenicity biomarkers	Secretome and proteome	Non-label short-GeLC, data collected in IDA and comparative quantitative analysis by SWATH-MS	PXD029377

One of the first studies applying a large-scale characterization of proteins in *B. xylophilus* focused on nematode surface coat (SC) proteins [99]. The nematodes SC is the outer layer of the cuticle that is recognized to have several different functions, from acting as an exoskeleton maintaining body morphology and integrity to other important roles in movement, growth, and osmoregulation. The cuticle surface interacts with the interior tissues of the plant host and it is known that the SC of plant parasitic nematodes has essential roles in host evasion, masking the surface cuticle to avoid host recognition and mitigating the defense response of host cells [65]. In this *B. xylophilus* proteomic study, Shinya and co-authors [99] found a group of proteins increased in nematodes grown in host pine seedlings compared to nematodes grown on the fungus *Botrytis cinerea*, by reversed-phase high-performance liquid chromatography (RP-HPLC) and SDS-PAGE. Identification of differentially expressed proteins was made by Matrix-assisted laser desorption ionization time-of-flight mass spectrometry analysis (MALDI-TOF); however, at this time, no protein information for *Bursaphelenchus xylophilus* was available in any database and so protein identification was made based on homolog or similar proteins in other organisms. Identified differential expressed proteins included several proteins possibly involved in the host immune response such as a regulator of reactive oxygen species (ROS) and two potential ROS scavengers, a glutathione S-transferase (GST) and a glyceraldehyde 3-phosphate dehydrogenase GAPDH [99].

Other primary proteomics study on *B. xylophilus* aimed to find molecular biomarkers specific to *B. xylophilus* in order to develop a more efficient detection method. Lee and co-authors applied several biochemical approaches followed by proteomic analysis by 2-DE nano-LC–MS/MS, to identify the target protein of a generated monoclonal antibody (MAb) specific to *B. xylophilus* and found that the antigenic target to that Mab was a galactose-binding lectin-1 (LEC-1) [100].

Later, protein markers for differentiating isolates of *B. xylophilus* were identified by studying differences among selected isolates by 2D-nano LC-Electrospray Ionization-MS/MS analysis quantitative proteomics (iTRAQ) [102], using *B. xylophilus* genomic data which became available by that time [53] for peptide identification. By using quantitative proteomics, it was possible to detect significant differences in protein regulation levels with high precision. As a result, 75 informative proteins were selected to be used as population-specific markers. Combined with a character compatibility method, a subset of 30 specific unique protein markers that allowed the compared classification of Iberian *B. xylophilus* isolates was identified [102].

From proteomics on *B. xylophilus*, the secreted proteins (secretome) have been of particular interest as they are directly involved in host-nematode interaction.

### *Bursaphelenchus xylophilus* secretome

**Fig. 3:**
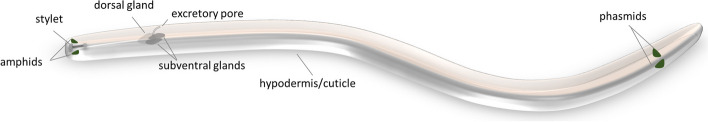
Illustration of secretory organs and natural openings in *Bursaphelenchus xylophilus*

The proteomics analysis focused on secreted proteins constitutes the secretome, representing all the proteins in the secretions. Secreted molecules are the hallmark of intercellular communication and mediate nematode-host interaction. In plant parasitic nematodes, directly interacting molecules include the surface coat molecules and secretions, from stylet or other natural openings, and constitute the most potential pathogenic molecules as they interact directly with host cells and cause disease. The main nematode organs producing secretions are the pharyngeal gland cells (two subventral gland cells and one dorsal gland cell), the hypodermis, which deposits secretions on the cuticle surface and the amphids. Additionally, at the tail end of the nematode there are phasmids that have a similar structure to the amphids and also produce secretions (Fig. 3). However, most of the molecules involved in parasitism are produced in the pharyngeal gland cells and are secreted into the host through the stylet [106].

*Bursaphelenchus xylophilus* secretome characterization became one of the main focuses when studying this nematode pathogenicity factors. The first complete profile of *B. xylophilus* secretome was achieved by Shinya et al. [101] by nano-LC/MS that identified a total of 1515 secreted proteins using *B. xilophilus* genomic data as database (PRJEA64437), including proteins involved in nutrient uptake, migration, and invasion from host defenses, potentially associated with *B. xylophilus* pathogenicity. The comparative functional analysis of secretome profiles among several plant-parasitic nematodes revealed a clear expansion of the number of peptidases and peptidase inhibitors in *B. xylophilus* secretome. Potential proteins that mimic host pine defense systems, such as two thaumatin-like proteins and one cysteine proteinase inhibitor, were also found in this secretome and could be a reflex of host-parasite co-evolution with native pine species in North America [101].

With the development of quantitative comparative proteomic methods, further studies on *B. xylophilus* secretomes were performed to highlight the most informative data. The identification of a set of putative most pathogenic proteins present in this nematode secretome has been accomplished by several authors during the last few years (Table 2). In 2016, a quantitative and comparative proteomic analysis of the secretome of *B. xylophilus* with the secretome of the closely related but non-pathogenic nematode, *B. mucronatus*, was performed by a short-GeLC approach, in combination with the SWATH-MS acquisition method for quantitative analysis. In this study, a higher number of secreted proteins (681) were identified in both nematode secretomes using a *B. xylophilus* (PRJNA192936) and *B. mucronatus* (PRJEB14884) transcriptomic-derived database than using *B. xylophilus* reference genome (PRJEA64437) (520), reflecting the importance of the use of the most adequate and complete database for peptide identification in proteomic studies. A total of 446 proteins were quantified in both nematode secretomes and from these 243 were found to be differentially regulated, with 158 proteins found increased in *B. xylophilus* secretome and 85 increased in *B. mucronatus* secretome. Functional features of these differentially regulated proteins suggested that differences in *B. xylophilus* and *B. mucronatus* pathogenicity to pine trees are mainly related to proteins associated with peptidase, glycosyl hydrolase and peptidase inhibitor activities (Table 2), which were found increased in *B. xylophilus* secretome compared to *B. mucronatus* secretome [103].

**Table 2:** Proteins identified as pathogenicity biomarkers in *Bursaphelenchus xylophilus* secretome. Common IDs in different studies are in italics. ^a^Correspondence determined by BLASTp analyses in this study

**Activity**	**Description**	**Original code**	**Best correspondence to *B. xylophilus*** **genomic data (PRJEA64437_WBPS17)**^**a**^			**References**
			**ID**	**% identity**	**E value**	
**Peptidase**	**Cysteine peptidase**	All_gs454_002631	BXY_0410100.1	100	0.00E + 00	[103]
		All_gs454_003203	BXY_0408100.1	100	1.10E-173	
		All_gs454_002316	BXY_0293600.1	99.5	4.00E-148	
		All_gs454_004450	BXY_0208100.1	100	1.40E-125	
		All_gs454_003244	BXY_0866900.1	100	4.00E-142	
		All_gs454_002475	BXY_0293300.1	96.4	0.00E + 00	
		BmPt2_003216	BXY_1098600.1	97.7	2.50E-129	
		BmPt2_000767	BXY_0208100.1 BXY_0208200.1	90.9	0.00E + 00	
		All_gs454_003032	*BXY_1052500.1*	99.7	0.00E + 00	
		BUX.s01288.15	*BXY_1052500.1*			[105]
		BUX.s00813.52	BXY_0618800.1			
		BXY_0101000.1				[51]
	**Serine peptidase**	All_gs454_001068	BXY_1709000.1	99.4	0.00E + 00	[103]
		All_gs454_005249	BXY_1770300.1	100	1.40E-144	
		All_gs454_005845	BXY_1121800.1	100	2.40E-127	
		All_gs454_000752	BXY_1545000.1	99.8	0.00E + 00	
		All_gs454_005600	BXY_0959000.1	100	3.80E-124	
		All_gs454_007198	BXY_1703500.1	100	1.70E-136	
		All_gs454_001272	BXY_1122100.1	98.8	0.00E + 00	
		All_gs454_001797	BXY_0963400.1	96.1	0.00E + 00	
		All_gs454_001410	BXY_1122200.1	100	0.00E + 00	
		BXY_0959000.1				[104]
		BXY_0963700.1				[51]
		BXY_1121700.1				
		BXY_1703500.1				
	**Metallo peptidase**	All_gs454_000155	BXY_1014700.1	98.6	0.00E + 00	[103]
		All_gs454_001243	BXY_1014200.1	99.6	0.00E + 00	
		All_gs454_002836	BXY_0363400.1	99.7	0.00E + 00	
		All_gs454_007821	BXY_0363400.1	100	5.20E-130	
		All_gs454_007450	BXY_0363400.1	100	2.20E-114	
		All_gs454_007798	BXY_0884000.1	100	1.80E-118	
	**Aspartic peptidase**	All_gs454_002706	BXY_0828700.1	96.8	0.00E + 00	[103]
		All_gs454_002182	BXY_1188300.1	97.4	0.00E + 00	
		All_gs454_002228	*BXY_0579700.1*	97.9	0.00E + 00	
		All_gs454_002143	BXY_1325300.1	98.1	1.00E-106	
		All_gs454_002300	BXY_1188000.1	98.2	0.00E + 00	
		BXY_0035000.1				[104]
		BXY_0555800.1				
		BXY_0820600.1				
		BXY_0821000.1				
		*BXY_0579700.1*				[51]
	**Threonine peptidase**	BmPt2_001890	BXY_1438300.1	99.2	5.90E-159	[103]
**Glycoside hydrolase**	**Chitinase**	All_gs454_002423	BXY_0052200.1	99.6	7.40E-167	[103]
		All_gs454_006276	BXY_1010600.1	98.2	1.00E-143	
		BmPt2_004053	BXY_1010600.1	90.4	7.50E-79	
		All_gs454_001611	BXY_1650900.1	99.8	0.00E + 00	
	**Cellulase (GH45)**	All_gs454_006369	BXY_0937900.1	100	2.60E-144	[103]
		BXY_1261000.1				[51]
	**Alpha-1,4-glucosidase**	All_gs454_000105	BXY_0176400.1	99.3	0.00E + 00	
	**Alpha galactosidase**	All_gs454_002135	BXY_0833500.1	99.5	0.00E + 00	
	**Fucosidase**	All_gs454_002563	BXY_0325000.1	97.8	1.40E-154	
	**Glucan endo-1,3-beta-D-glucosidase**	All_gs454_005432	BXY_0535400.1	99.2	0.00E + 00	
	**Glycosyl ceramidase (GH30)**	BUX.s00713.1066	BXY_0413000.1	100	0.00E + 00	[105]
**Other hydrolase**	**Acid sphingomyelinase**	BXY_0542900.1				[104]
	**Histidine acid phosphatase**	BXY_1236200.1				
	**Lysozyme-like protein (GH18)**	BXY_0522000.1				
	**Trehalase (GH37)**	BXY_1306200.1				
	**Lipase**	BXY_1125700.1				
		BXY_0707300.1				[51]
		BXY_0824600.1				
		BUX.s00961.62	BXY_0630900.1	100	0.00E + 00	[105]
**Endopeptidase inhibitor**	**Serine type**	All_gs454_001641	BXY_0363400.1	100	0.00E + 00	[103]
	**Cysteine-type**	All_gs454_009328	BXY_0816900.1	97.7	5.10E-82	
		All_gs454_014827	BXY_1510800.1	100	1.40E-57	
		All_gs454_008917	BXY_0510100.1	100	1.40E-65	
**Oxido reductase**	**Short-chain dehydrogenase/reductase**	BXY_0328000.1				[104]
	**γ-interferon-inducible lysosomal thiol reductase**	BXY_0504300.1				[51]
**Diverse**	**Signal recognition particle**	BXY_1012800.1				[104]
	**Intermediate filament tail domain protein**	BXY_1639600.1				
	**Integral component of membrane**	BXY_0888500.1				
	**C-type Lectin**	BXY_0360300.1				
	**Degenerin unc-8**	BXY_1546700.1				
	**Structural maintenance of chromosomes protein**	BXY_1747100.1				
	**Venom allergen-like protein**	BXY_1378500.1				[51]
**Putative proteins with no description**		BXY_0073000.1				[104]
		BXY_1760900.1				
		BXY_0799700.1				
		BXY_0583800.1				
		BXY_0463500.1				
		BXY_0927300.1				[51]
		BXY_0174200.1				
		BXY_0073000.1				

Later, the secretomes of *B. xylophilus* under the stimuli of pine species with different kinds of susceptibility to PWN, *P. pinaster*, as high susceptible, and *P. pinea*, as low susceptible, were also compared using the same methodology. Quantitative differences among the 776 proteins detected in these secretomes, highlighted diverse responses from the nematode to overcome host defenses with different susceptibilities. Functional analyses of the 22 proteins found increased in the nematode secretome under *P. pinaster* stimuli revealed that proteins with peptidase, hydrolase, and antioxidant activities were the most represented [104].

In a semi-quantitative proteomic study (3D-protein separation system used for comparative and semi-quantitative proteome analysis), the comparative secretome analysis among four *B. xylophilus* isolates with different levels of virulence has been carried out and four candidate virulence determinants identified: one lipase, two cysteine peptidases, and glycoside hydrolase family 30 [105].

In 2022, short-GeLC/SWATH-MS was used to perform a deep characterization of proteomic changes across two *B. xylophilus* isolates with different virulence and in different conditions, pine extract (PE) and fungus stimuli. From the 1456 proteins identified in the secretomes of both isolates, 13 proteins were found increased in *B. xylophilus* virulent isolate secretome: five peptidases, one cellulase (GH45), two lipases, a γ-interferon-inducible lysosomal thiol reductase (GILT) and other three putative proteins with no description and associated to *B. xylophilus* virulence. Moreover, from the proteome analysis of both isolates in PE and fungus, 30 proteins were selected as putatively related to more virulence, mainly related to peptidase, cellulase, cytochrome P450 and oxidoreductase activities [51]. Interesting, a recent functional characterization of one of *B. xylophilus* lipases (BXY_0824600.1) selected as pathogenicity biomarkers showed its interaction with two class I chitinases from the host tree and its essential role on the virulence of this nematode [107].

**Fig. 4:**
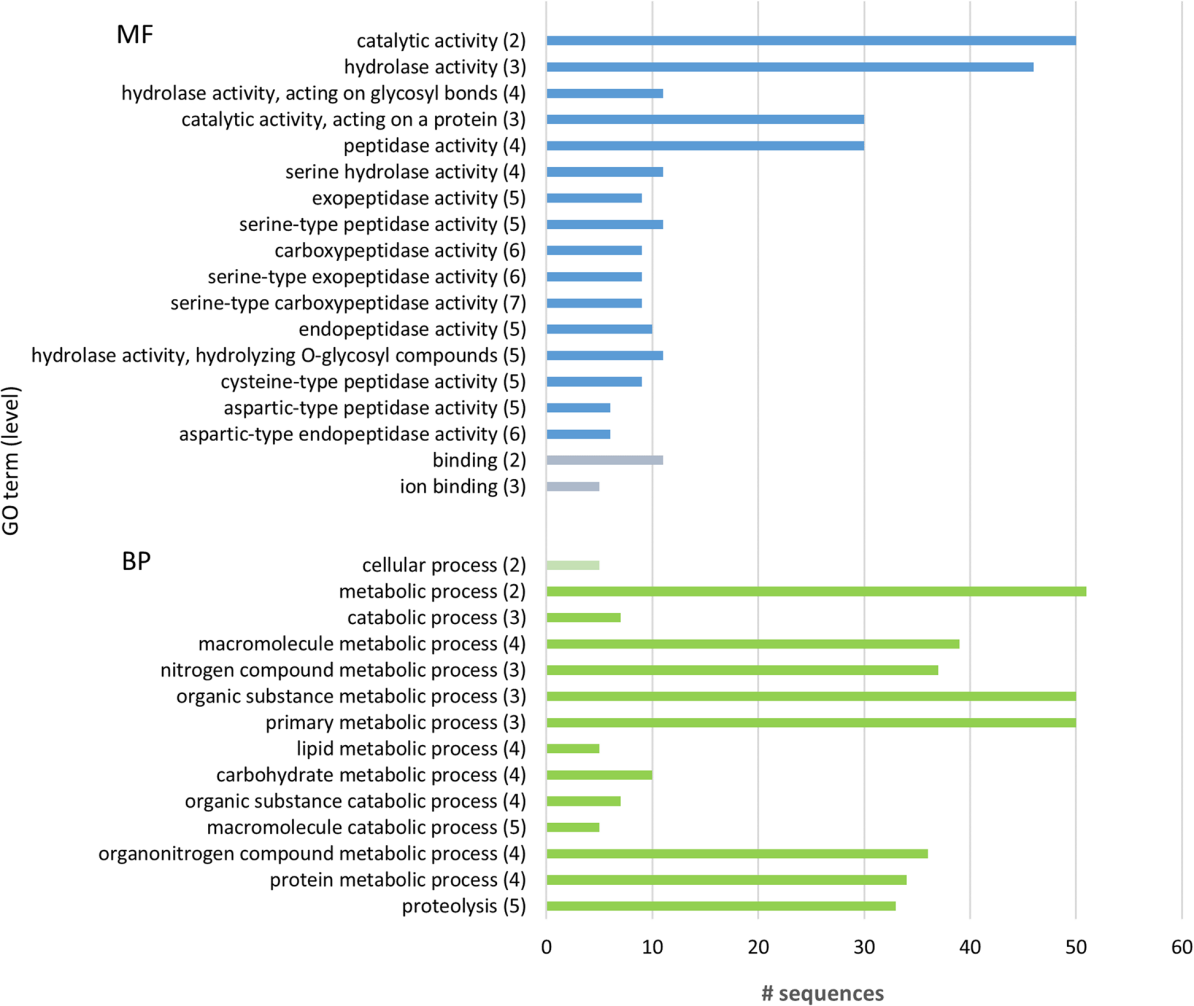
Functional annotation of protein sequences correspondent to selected pathogenicity biomarkers in *Bursaphelenchus xylophilus* secretome. Number of sequences associated to each gene ontology (GO) term at molecular function (MF) and biological process (BP) GO category

Gene ontology (GO) annotation of protein sequences correspondent to selected pathogenicity biomarkers in *B. xylophilus* secretome (Table 2) was performed in this study using Blast2GO [108] from OmixBox [109] to meet a global idea of the functions associated to these proteins. The majority are proteins associated to hydrolase activity in molecular function GO category, namely hydrolases acting on glycosyl bonds and peptidase activities, and also associated to metabolic processes GO terms in biological process category, such as organic substance metabolic process and proteolysis (Fig. 4). Overall, the proteins identified as putative virulence biomarkers belong to groups of proteins whose activities could be associated with invasion, migration and degradation of host tissues, protection of the nematode and suppression of host defenses.

### Integrated analysis of *Bursaphelenchus xylophilus* biomarkers identified by proteomics

The presented proteomic studies mainly used the *B. xylophilus* genomic data derived from Bioproject PRJEA64437 for protein identification. However, the first data available for deduced protein sequences (PRJEA64437_WS24), which is no longer publicly available, used different codes than the protein sequences PRJEA64437_WBPS17, available from Wormbase Parasite. In order to compare and better integrate the results obtained from the different proteomic studies, a local BLASTp [110] of one set of data against the other was performed in this study to find the best correspondence among the different protein codes and the possible similarity of the several biomarkers identified in the different studies. Additionally, the best correspondence of transcriptomic-derived protein identifications (PRJNA192936 and PRJEB14884) to *B. xylophilus* genomic data PRJEA64437_WBPS17 was also performed. With this analogy, several putative virulence biomarkers were common among the different *B. xylophilus* secretomic studies (Table 2).

In addition, finding the best correspondence of the 75 proteins identified as informative as population-specific markers by Ciordia et al. [102] revealed that most of these were also present in secretome profiles presented in several secretome studies (Table 3), revealing that the majority of population markers are secreted proteins. From these, four of them correspond to proteins selected as putative virulence biomarkers presented in Table 2: a serine peptidase (BXY_1703500.1); a metallo peptidase (BXY_1014700.1); an alpha-galactosidase (BXY_0833500.1) and a endopeptidase inhibitor (BXY_0816900.1) and nine were also highlighted in transcriptomic based studies [60, 111–113] as putative pathogenicity related proteins (Table 3). Interestingly the metallo peptidase BXY_1014700.1, besides being selected as a putative virulence biomarker from the *B. xylophilus* vs *B. mucronatus* secretomes comparison, it was also referred as a putative effector found to be highly upregulated during infection [57] and present in *B. xylophilus* transcriptome at several infection stages [60].

**Table 3:** *Bursaphelenchus xylophilus* putative population-specific markers and their mention in secretomic and transcriptomic studies. IDs selected as putative virulence biomarkers in presented secretomic studies are marked in bold. ^a^Correspondence determined by BLASTp analyses in this study

**Number and accession ID from** **Ciordia et al. 2016**		**Best correspondence to *B. xylophilus* genomic data (PRJEA64437_WBPS17)**^***a***^			**Presence in *B. xylophilus* secretome- References**	**Presence in *B. xylophilus* transcriptome as related to pathogenicity- References**	**Description**
		**ID**	**% identity**	**E value**			
3	BUX.c03212.1	BXY_1447300.1	100	2.27E-98	[51, 104]		Pecanex-like protein
4	BUX.c04300.1	BXY_1507900.1	100	4.18E-97	[51]		EGF-like domain-containing
9	BUX.s00036.143	BXY_0436400.1	100	7.10E-94	[51]		40S ribosomal protein S24
10	BUX.s00036.52	BXY_0427900.1	100	1.13E-118			Aspartic peptidase
11	BUX.s00055.288	BXY_0161100.1	100	1.17E-125	[51, 103, 104]		Ferritin
13	BUX.s00083.14	BXY_0840600.1	100	0.00E + 00	[51]		Carboxylesterase
21	BUX.s00083.88	BXY_0847200.1	99.6	0.00E + 00			Oxidored FMN domain-containing protein
22	BUX.s00083.91	BXY_0847500.1	100	0.00E + 00	[51]		Oxidored FMN domain-containing protein
24	BUX.s00110.21	BXY_0540200.1	100	7.52E-62	[103]		Saposin B-type domain-containing protein
26	BUX.s00110.84	BXY_0545400.1	100	4.16E-113			Glutathione S-transferase
29	BUX.s00116.187	BXY_0752000.1	100	6.33E-106			Unknown
30	BUX.s00116.330	BXY_0765700.1	100	0.00E + 00	[51, 103]		Proactivator polypeptide
31	BUX.s00116.358	BXY_0768100.1	100	0.00E + 00	[51, 104]		Inorganic diphosphatase
32	BUX.s00116.517	BXY_0783200.1	100	0.00E + 00	[51, 104]		Aldo-ket reductase
38	BUX.s00116.871	**BXY_0816900.1**	100	2.23E-78	[51, 103, 104]		Endopeptidase inhibitor cysteine-type
42	BUX.s00119.22	BXY_0935900.1	100	1.29E-147	[51]		TspO/MBR-related protein
46	BUX.s00139.156	BXY_0592900.1	100	4.76E-133	[51, 104]		Translationally-controlled tumor protein
47	BUX.s00139.22	BXY_0580100.1	100	0.00E + 00	[51, 103, 104]	[112]	Unknown
48	BUX.s00139.23	BXY_0580200.1	100	0.00E + 00	[51, 103, 104]		Secreted protein
49	BUX.s00139.24	BXY_0580300.1	100	0.00E + 00	[51, 103]		Unknown
54	BUX.s00150.2	BXY_1230900.1	100	2.53E-112			GLOBIN domain-containing protein
57	BUX.s00252.68	BXY_0649500.1	92.2	3.50E-71	[51, 101, 103, 104]		Beta-hexosaminidase
58	BUX.s00252.81	BXY_0650500.1	100	0.00E + 00	[51, 104]		Hydroxymethylglutaryl-CoA synthase
59	BUX.s00298.18	BXY_0555900.1	100	0.00E + 00	[51, 103, 104]		Aspartic peptidase
61	BUX.s00298.19	BXY_0556000.1	100	0.00E + 00	[51, 103, 104]		Unknown
62	BUX.s00298.7	BXY_0554700.1	100	0.00E + 00			Unknown
63	BUX.s00333.150	BXY_0610200.1	100	2.60E-79	[51, 103, 104]		SnoaL-like domain-containing protein
67	BUX.s00333.63	BXY_0601700.1	97.9	2.77E-134			Unknown
75	BUX.s00351.347	BXY_1544500.1	100	3.66E-87	[101]	[111]	Cysteine peptidase inhibitor
76	BUX.s00351.387	BXY_1548700.1	100	0.00E + 00	[51]		Aspartic peptidase
82	BUX.s00397.93	BXY_0701300.1	100	7.09E-115	[51, 104]		60S ribosomal protein L24
86	BUX.s00422.677	BXY_1672800.1	100	3.82E-122	[51, 104]	[112]	Unknown
99	BUX.s00508.71	BXY_0923300.1	99.6	0.00E + 00	[51]		Aminopeptidase
102	BUX.s00579.159	BXY_1386500.1	100	0.00E + 00	[51, 104]	[60]	Catalase
106	BUX.s00613.1	BXY_1265900.1	100	3.03E-52	[51]		CsbD family protein
110	BUX.s00647.122	BXY_0299600.1	100	6.21E-155	[51, 101, 103, 104]		Glutathione s-transferase
113	BUX.s00649.27	BXY_0967200.1	100	0.00E + 00	[51, 103, 104]		PKS ER domain-containing protein
120	BUX.s00713.693	BXY_0376300.1	100	0.00E + 00			Unknown
121	BUX.s00713.744	BXY_0381200.1	100	0.00E + 00	[51, 103, 104]		Glycoside hydrolase 35
123	BUX.s00713.89	BXY_0317400.1	100	0.00E + 00			Serine/threonine-protein phosphatase
124	BUX.s00713.926	BXY_0399900.1	100	0.00E + 00	[51, 104]	[113]	Short-chain dehydrogenase
126	BUX.s00713.953	BXY_0402700.1	95.5	0.00E + 00		[111, 112]	Aspartic peptidase
127	BUX.s00713.955	BXY_0402900.1	99.5	1.38E-162			Aspartic peptidase
129	BUX.s00729.2	BXY_1201500.1	99.7	0.00E + 00			Aspartic peptidase
134	BUX.s00961.41	BXY_0629000.1	100	2.95E-153	[51, 103]		Glutathione S-transferase
137	BUX.s01038.263	BXY_0192700.1	100	0.00E + 00	[51, 103, 104]		Unknown
138	BUX.s01038.84	BXY_0176900.1	100	3.04E-54	[51]		Small nuclear ribonucleoprotein G
140	BUX.s01063.193	BXY_0503400.1	100	9.03E-104	[51, 103, 104]	[112]	Transthyretin-like family protein
141	BUX.s01063.30	BXY_0488400.1	100	0.00E + 00	[51]		Unknown
148	BUX.s01092.201	BXY_0070300.1	100	7.11E-95	[103]		Major sperm protein
150	BUX.s01092.9	BXY_0052900.1	100	6.24E-105	[51, 101]		Thioredoxin-like protein
152	BUX.s01109.342	BXY_1742300.1	100	0.00E + 00	[51, 103, 104]		Galectin
154	BUX.s01109.576	BXY_1765200.1	100	2.29E-64			Prothymosin alpha-like protein
155	BUX.s01109.624	BXY_1769700.1	100	2.89E-114	[51, 101, 103, 104]		Superoxide dismutase
159	BUX.s01143.144	BXY_0228700.1	100	0.00E + 00	[51, 104]	[113]	Oxido reductase
164	BUX.s01144.22	BXY_0103500.1	100	0.00E + 00	[51, 103, 104]		Unknown
168	BUX.s01147.118	BXY_0203300.1	100	0.00E + 00	[51, 103, 104]		Calreticulin
169	BUX.s01147.119	BXY_0203300.1	97.5	5.13E-70			Calreticulin
172	BUX.s01147.198	BXY_0210100.1	100	0.00E + 00	[51]		Alcohol dehydrogenase
179	BUX.s01254.196	BXY_1692800.1	100	1.54E-122	[51, 103, 104]		Unknown
180	BUX.s01254.217	BXY_1694500.1	100	0.00E + 00			Arginine kinase
182	BUX.s01254.306	**BXY_1703500.1**	100	0.00E + 00	[51, 103, 104]		Serine peptidase
183	BUX.s01254.317	BXY_1704600.1	100	0.00E + 00			RNA binding protein
185	BUX.s01259.90	**BXY_0833500.1**	100	0.00E + 00	[51, 103, 104]		Alpha-galactosidase
187	BUX.s01268.18	BXY_0957900.1	100	2.26E-87			Heat shock protein
189	BUX.s01268.84	BXY_0964200.1	100	0.00E + 00			ADP/ATP translocase
191	BUX.s01281.111	BXY_1566800.1	98.6	2.43E-153	[51, 104]		DUF2147 domain-containing protein
195	BUX.s01281.502	BXY_1602400.1	100	6.86E-129	[51, 104]		Haloacid dehalogenase-like hydrolase
197	BUX.s01281.82	BXY_1564300.1	100	0.00E + 00	[51, 101, 103, 104]		Aspartic peptidase
198	BUX.s01438.101	BXY_0665500.1	100	0.00E + 00	[51, 103, 104]		S-formylglutathione hydrolase
202	BUX.s01513.79	BXY_0259100.1	100	8.45E-77	[51, 103, 104]		MSP domain-containing protein
205	BUX.s01653.173	BXY_0016100.1	98.6	5.46E-163	[51, 104]		GFO IDH MocA domain-containing protein
206	BUX.s01653.174	BXY_0016100.1	100	9.80E-78	[51, 104]		GFO IDH MocA domain-containing protein
210	BUX.s01656.23	BXY_0873400.1	100	4.28E-156			NADH dehydrogenase
212	BUX.s01661.67	**BXY_1014700.1**	100	0.00E + 00	[51, 103, 104]	[57, 60]	Metallo peptidase-neprilysin

### Host trees proteomics

Few studies have focused on proteomic comparison of host trees with different susceptibility to PWD. Proteomic differences between two *P. massoniana* provenances inoculated with PWN revealed the presence of proteins involved in hydrogen peroxide scavenging capacity protecting the redox homeostasis system associated with resistance [114]. Another proteomic study focused on resistant clones of *P. massoniana* inoculated with PWN, showed highly expressed aspartic proteases suggesting the capacity of these trees to degrade nematode-related proteins [115]. Proteomics on pine trees is still understudied but is a promising strategy to better understand resistance mechanisms involved in PWD, which needs further exploration.

## Conclusions and future perspectives

Presently, proteomics constitutes priority research for any organism, since the number of protein species differs from the number of genes and transcripts, approaching the phenotype more than the genotype. Nowadays proteomics approaches are massively dependent on mass spectrometry techniques. These instruments are getting faster, more sensitive and with a higher dynamic range which, combined with different sample fractionation strategies, allows an even deeper proteome coverage. The increase in the quantity and quality of the data is being followed by an increase in the available tools to process the data. Proteomics on PWD, not only provides a molecular knowledge of the mechanisms associated to disease development and resistance, but also allows the identification of key proteins (biomarkers) and their possible interaction between the involved species. Targeted proteomics, a mass spectrometry-based protein quantification technique with high sensitivity, accuracy, and reproducibility, may be a powerful technique that could be useful in the future as a method to detect identified biomarkers, useful for the development of new PWD control measures.

**Fig. 5:**
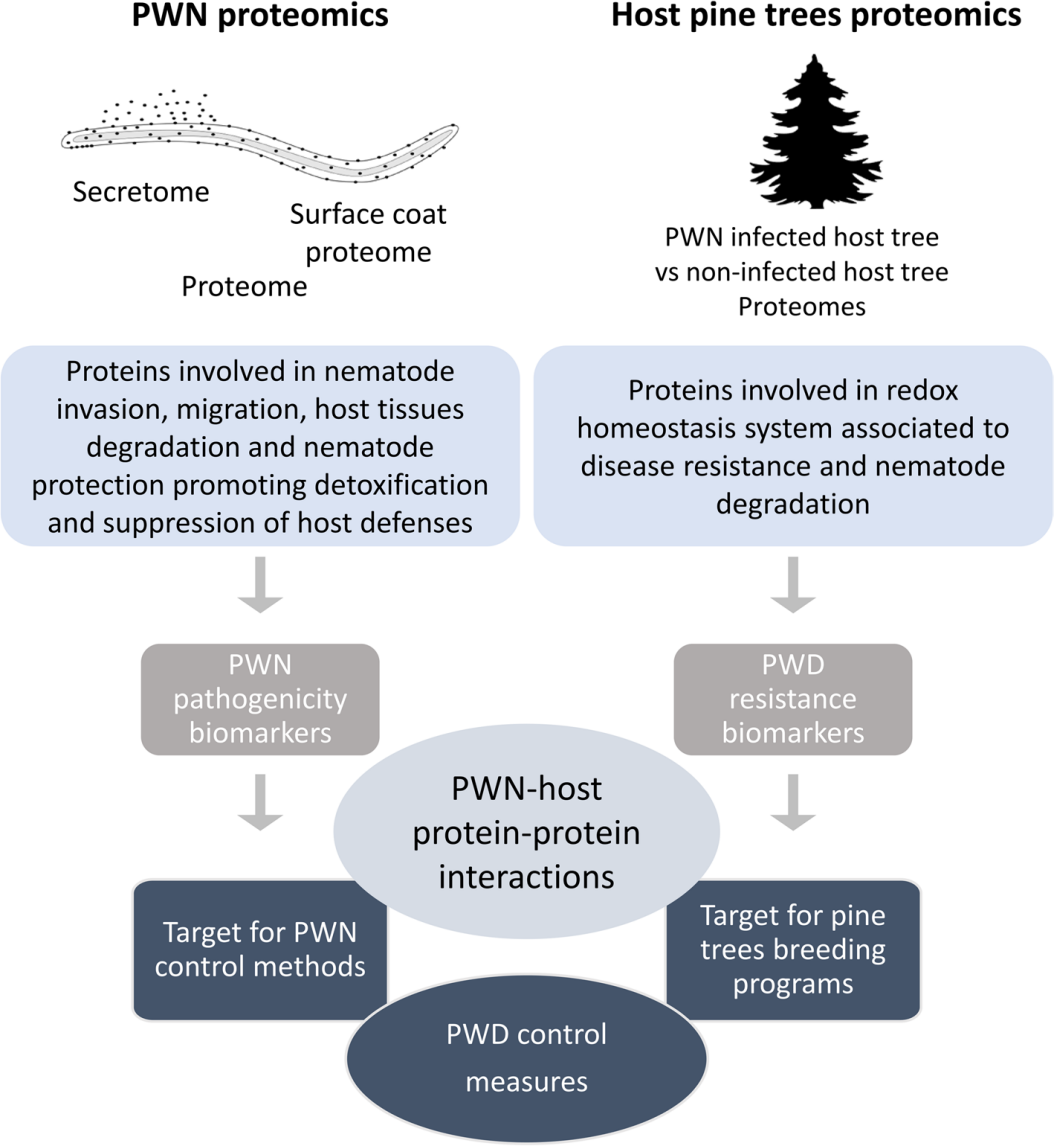
Major outcomes of proteomics research on pine wilt disease (PWD). Pinewood nematode (PWN)

Highlighted proteins in the different proteomic studies on PWD, and compiled in this review, functioned in different ways important to *B. xylophilus* infection and survival, such as breaking down host cell walls, promoting feeding efficiency, suppressing host defenses, promoting detoxification, and thus playing virulence functions. Moreover, host tree proteomics revealed the presence of proteins involved in the redox homeostasis system associated to resistance and aspartic proteases to degrade nematode-related proteins. Besides contributing to the clarification of the mechanisms implicated in PWN pathogenicity and host resistance, this information is usefulness for developing new control strategies for this important forest pest such as the development of new nematicidal compounds or molecular based control strategies like host-induced gene silencing and identification of pine resistance markers that could be used in breeding programs (Fig. 5). However, the molecular mechanisms involved in *B. xylophilus* pathogenicity and host resistance and *B. xylophilus* adaptation to different hosts under different climate conditions should be further explored. Integration of proteomics of PWN and host trees with physiological, biochemical, and other large-scale omics provides a comprehensive understanding of the different biological processes involved in this interaction, from growth and development to responses to biotic and abiotic stresses. With global climate change, differences in host pines susceptibility to PWN infection are expected and thus, clarifying the molecular mechanisms associated with these differences in host susceptibility/resistance and identification of pine proteins, which could be used as markers in breeding programs, will be essential to the development of new control strategies and more sustainable management of pine forests.

## Data Availability

All data generated or analysed during this study are included in this published article.
